# Nucleolin promotes Ang II‐induced phenotypic transformation of vascular smooth muscle cells by regulating EGF and PDGF‐BB

**DOI:** 10.1111/jcmm.14888

**Published:** 2020-01-01

**Authors:** Li Fang, Kang‐Kai Wang, Peng‐Fei Zhang, Tao Li, Zhi‐Lin Xiao, Mei Yang, Zai‐Xin Yu

**Affiliations:** ^1^ Department of Cardiology Xiangya Hospital Central South University Changsha China; ^2^ Department of Cardiology The First Hospital of Changsha Changsha China; ^3^ Department of Pathophysiology Xiangya School of Medicine Central South University Changsha China; ^4^ Key Laboratory of Cancer Proteomics of Chinese Ministry of Health Xiangya Hospital Central South University Changsha China; ^5^ Department of Geriatric Cardiology Xiangya Hospital Central South University Changsha China

**Keywords:** angiotensin II, EGF, nucleolin, PDGF‐BB, phenotypic transformation, vascular smooth muscle cells

## Abstract

RNA‐binding properties of nucleolin play a fundamental role in regulating cell growth and proliferation. We have previously shown that nucleolin plays an important regulatory role in the phenotypic transformation of vascular smooth muscle cells (VSMCs) induced by angiotensin II (Ang II). In the present study, we aimed to investigate the molecular mechanism of nucleolin‐mediated phenotypic transformation of VSMCs induced by Ang II. Epidermal growth factor (EGF) and platelet‐derived growth factor (PDGF) inhibitors were used to observe the effect of Ang II on phenotypic transformation of VSMCs. The regulatory role of nucleolin in the phenotypic transformation of VSMCs was identified by nucleolin gene mutation, gene overexpression and RNA interference technology. Moreover, we elucidated the molecular mechanism underlying the regulatory effect of nucleolin on phenotypic transformation of VSMCs. EGF and PDGF‐BB played an important role in the phenotypic transformation of VSMCs induced by Ang II. Nucleolin exerted a positive regulatory effect on the expression and secretion of EGF and PDGF‐BB. In addition, nucleolin could bind to the 5′ untranslated region (UTR) of EGF and PDGF‐BB mRNA, and such binding up‐regulated the stability and expression of EGF and PDGF‐BB mRNA, promoting Ang II‐induced phenotypic transformation of VSMCs.

## 1 INTRODUCTION

Nucleolin is the most abundant RNA‐binding protein in eukaryotic nucleus. It has many biological functions, and its main functions include the regulation of ribosomal RNA biosynthesis and ribosome assembly.^1^, ^2^ Recent studies have shown that nucleolin is also involved in physiological and pathological processes, such as cell growth, proliferation, apoptosis, cytokinesis and inflammatory immunity.^3^, ^4^, ^5^ Studies have shown that nucleolin is highly expressed in rapidly proliferating tissues and cells, including stem cells and tumour cells, and it promotes stem cell renewal and tumour cell growth.^6^, ^7^, ^8^ The expression level of nucleolin is positively correlated with the rate of cell division. Although the expression of nucleolin in tumours and other rapidly dividing cells is quite high, its expression remains very low in non‐dividing cells. Therefore, nucleolin, as an effective marker, is often used to determine the extent of cell proliferation.^9^ Phenotypic transformation of vascular smooth muscle cells (VSMCs) is a critical initial step in the proliferation and migration of VSMCs. Our previous studies have indicated that the expression of nucleolin at the mRNA and protein levels is gradually increased after VSMCs are stimulated with angiotensin II (Ang II) at different concentrations and durations, and Ang II can induce nucleolin translocation from nucleus to cytoplasm. Such findings further confirm that nucleolin plays a positive regulatory role in Ang II‐mediated phenotypic transformation of VSMCs.^10^ However, how the cellular nucleolin promotes phenotypic transformation of VSMCs, as well as its specific molecular mechanism, remains largely undetermined.

A large number of studies have revealed that the RNA‐binding properties of nucleolin play a fundamental role in a variety of biological functions, and the specific nucleic acid‐binding element is ‘(T/G) CCC G (A/G)’.^6^, ^11^, ^12^, ^13^, ^14^, ^15^ Therefore, we first analysed the mRNA sequences of several VSMC phenotype transformation genes by bioinformatics analysis and found that the mRNA sequences of 12 VSMC phenotype‐related genes, such as epidermal growth factor (EGF) and platelet‐derived growth factor (PDGF), contained different amounts of nucleolin‐specific binding element (Table 1). Based on our previous findings, we hypothesized that Ang II‐induced up‐regulation of nucleolin, and then it bound to the mRNA of EGF and PDGF through the RNA‐binding properties of nucleolin, and regulated the stability of EGF and PDGF mRNA, thus positively regulating the phenotypic transformation. In the present study, we used Ang II to induce phenotypic transformation of VSMCs, and the regulatory role of nucleolin in the phenotypic transformation of VSMCs was identified by molecular biology methods, such as nucleolin gene mutation, gene overexpression, RNA interference technology and protein‐mRNA interaction analysis (post‐transcriptional level). Moreover, we assessed the protein‐RNA interaction to elucidate the molecular mechanism underlying the regulatory effect of nucleolin on phenotypic transformation of VSMCs. Collectively, our study provided valuable insights into the regulatory mechanism of VSMC phenotypic transformation and offered a new strategy for controlling and reversing the phenotypic transformation of VSMCs.

**Table 1:** Primer sequences and RNA probes used in the present study

Genes	Sequences
Epidermal growth factor (388 bp)	Forward: 5‐ CCGCTCGAGCAAAAGGAGAAGCCATCAGGG ‐3
	Reverse: 5′‐ CCCAAGCTTGGTCTCGGTGGTTCTAAGGT ‐3′
Platelet‐derived growth factor‐BB (383 bp)	Forward: 5′‐CGAGTTGGACCTGAACATGA ‐3′
	Reverse: 5′‐CAGCTGCCACTGTCTCACAC ‐3′
β‐actin (228 bp)	Forward: 5′‐CCTCGCCTTTGCCGATCC‐3′
	Reverse: 5′‐GGATCTTCATGAGGTAGTCAGTC‐3′
Epidermal growth factor (42 bp)	Forward: 5′‐ GGGGCCGGGGGCGGCG GCGCCCGGGGGC CATGC GGGTGAGCC‐3′‐ biotin‐labelled
	Reverse: 5′‐GGCTCACCCGCATGGCCCCCGGGCGCCGCCGCCCCC GGCCCC‐3′‐ biotin‐labelled
Platelet‐derived growth actor‐BB (42 bp)	5′‐TTTAGCCCCATCCCTCATTCCCGGTGGGGTTTGGAACTTTCC‐3′‐ biotin‐labelled
	5′‐GGAAAGTTCCAAACCCCACCGGGAATGAGGGATGGGGCTAAA‐3′‐ biotin‐labelled

## 2 MATERIALS AND METHODS

### 2.1 Cell culture and treatment

Vascular smooth muscle cells were purchased from Shanghai Tiancheng Life Technologies (ATCC, No. CRL‐1476) and maintained in DMEM supplemented with 10% heat‐inactivated foetal bovine serum (FBS), 2 mmol/L glutamine and antibiotic‐antimycotic mixture in a humidified atmosphere containing 5% CO_2_ and 95% air. VSMCs were stimulated with Ang II at different concentrations (10^−8^, 10^−7^, 10^−6^ and 10^−5^ mmol/L) for 48 hours or 10^−5^ mmol/L Ang II for different durations.

### 2.2 Bioinformatics analysis of nucleolin‐binding elements

The mRNA sequences of 12 VSMC phenotype‐associated genes, such as epiregulin, were searched against PubMed (http://ncbi.nlm.nih.gov/pubmed/) and UCSC Genome Browser gene databases (http://genome.ucsc.edu/), and the mRNA sequences of these genes were used to screen nucleolin‐binding elements, such as ‘(T/G) CCCG (A/G)’ and analyse whether these binding elements were located in the 5′ UTR, 3′ UTR or coding region.

### 2.3 Extraction of total cell proteins

After corresponding treatment, VSMCs were washed with pre‐cooled PBS for three times. According to the cell confluence, 50‐80 µL 12× SDS lysis buffer (100 mmol/L Tris‐HCl, 200 mmol/L DTT, 40 g/L SDS, 20% glycerol; pH 6. 8) was added to each well. Subsequently, the cell lysates were transferred into the 1.5‐mL centrifuge tubes, denatured at 100°C for 10 minutes and then centrifuged at 12 000 *g* for 10 minutes at 4°C. The supernatant was transferred to a 0.5‐mL centrifuge tube and stored at −70°C. The protein concentration was determined using a Bicinchoninic Acid (BCA) Protein Assay kit (Shanghai Biyuntian Biotechnology, Ltd.) according to the manufacturer's instructions.

### 2.4 Reverse transcription‐quantitative polymerase chain reaction (RT‐qPCR)

Total RNA was isolated using the Rneasy kit (Qiagen) according to the manufacturer's instructions, and 2 μg purified RNA was reversely transcribed into cDNA using oligo(dT) primers. The expressions of target genes were examined on a 7500 Fast Real‐Time PCR system (Applied Biosystems) using a QuantiTect SYBR Green PCR Kit (Qiagen). Briefly, after an initial denaturation step at 95°C for 10 seconds, amplifications were carried out with 40 cycles at a melting temperature of 95°C for 5 seconds and an annealing temperature of 60°C for 30 seconds. Primer sequences used in the present study were listed in Table 1. The specificity of amplification was verified by melting curve analysis and validated by electrophoresis on agarose gels. The relative expressions of target genes were calculated by 2^−ΔΔCt^ method, and β‐actin was selected as the housekeeping gene. Each experiment was conducted in triplicate.

### 2.5 Amplification and extraction of recombinant plasmids

The recombinant plasmids of pcDNA3.1‐Nuc, PsiRNA‐Nuc and Nuc1‐309 were kindly gifted by Professor Kangkai Wang, Department of Pathophysiology, Xiangya School of Medicine, Central South University. Nucleolin expression plasmid (pcDNA3.1‐Nuc), RNA interference fragment of nucleolin (PsiRNA‐Nuc) and Nuc1‐309 [a nucleolin mutant lacking a carboxy terminus (Nuc310‐713), ie the amino acid containing the RNA‐binding domain (RBD) was deleted] were transformed into competent cells, and then monoclonal colonies were inoculated into 5 mL LB medium containing corresponding antibiotics and maintained at 37°C on a rotary bed (250 rpm) overnight. Subsequently, the culture suspension was transferred to 200 mL LB medium containing the corresponding antibiotics. After the turbidity reached the standard, the bacteria were collected for the extraction of plasmids. PsiRNA‐Nuc, pcDNA3.1‐Nuc and Nuc1‐309 were extracted by Plasmid Extraction Kit according to the manufacturer's instructions, and the DNA concentration of purified plasmids was determined by spectrophotometer. Finally, isolated plasmids were stored at −70°C.

### 2.6 Transient transfection

Transfection of cells was carried out following the manufacturer's instructions (MegaTran 1.0; OriGene). Briefly, 5 × 10^5^ cells were grown in 5 mL appropriate complete growth medium at 37°C in a CO_2_ incubator until the cells reached 70%‐80% confluence (24 hours). After washed with serum‐free and antibiotic‐free medium, the cells were transfected with pcDNA3.1‐Nuc/psiRNA‐Nuc (experimental), pcDNA3.1/psiRNA (vector control) or Nuc1‐309 by mixing with 6 μL MegaTran 1.0 containing 2 μg DNA, and the mixture was placed at room temperature for about 10 minutes. Subsequently, the mixture was added to a 6‐well plate, followed by gentle agitation and incubation at 37°C for 24 hours in a CO_2_ incubator.

### 2.7 Western blotting analysis

Following various treatments, VSMCs cells were lysed with radioimmunoprecipitation assay (RIPA) buffer (Shanghai Biyuntian Biotechnology, Ltd.). The protein concentration was determined using BCA assay. Whole‐cell lysates were subjected to SDS‐PAGE and then transferred onto polyvinylidene fluoride (PVDF) membranes. The blots were incubated with respective primary antibodies against nucleolin (rabbit polyclonal antibody, Sigma), α‐SM‐actin (mouse monoclonal antibody, Boster Biotech), SM22a (rabbit polyclonal antibody, Abcam), calponin (mouse monoclonal antibody, Abcam), OPN, EGF, PDGF‐BB (rabbit polyclonal antibody, Abcam) and β‐actin (mouse monoclonal antibody, Abcam) at 25°C for 2 hours. Subsequently, the blots were incubated with peroxidase‐conjugated secondary antibodies at 25°C for 1 hours. Immunoreactive bands were visualized utilizing enhanced chemiluminescence detection kit (Beyotime Institute of Biotechnology) according to the manufacturer's instructions, and the densitometry analysis was performed by scion image software.

### 2.8 Enzyme‐linked immunosorbent assay (ELISA)

Levels of EGF and PDGF‐BB in the culture medium were determined by commercially available rat ELISA kits for EGF and PDGF‐BB (Abcam) according to the manufacturer's instructions. Considering that EGF and PDGF‐BB are cell secreting proteins, the cells were cultured in a 96‐well plate for 48 hours. When the cells reached 80%‐90% confluence, the serum‐free medium was replaced before adding Ang‐II. Cell culture supernatant was collected after Ang II challenge at different concentrations and durations, and 200 μL supernatant was added into a 96‐well plate pre‐coated with the corresponding mAbs of EGF and PDGF‐BB and incubated at 37°C for 2 hours. Subsequently, liquid was removed, and 200 μL enzyme‐labelled secondary antibody was added into each well, followed by incubation at 37°C for 2 hours. Wells were washed with TBS (0.01 mmol/L) for three times (3 minutes for each wash) and then incubated with substrate solution (200 μL) at 25°C for 30 minutes. Next, the stop solution was added to terminate the reaction. The optical density (OD) was measured by an enzyme‐labelled instrument (Startfax 2100) at a wavelength of 450 nm. The sample concentration was calculated by linear equation.

### 2.9 Co‐immunoprecipitation of nucleolin protein and EGF, PDGF‐BB mRNA

After treatment, VSMCs were homogenized in 1 mL RIPA buffer containing protease inhibitors (10 µL/0.1 g tissue weight; Sigma‐Aldrich; Merck KGaA). Soluble proteins were collected after centrifugation at 12 000 *g* for 15 minutes at 4°C. After quantitative analysis by BCA method, protein supernatant was divided into three equal fractions (500 μL of each) as follows: one was used as ‘Input’ sample; one was used as control IgG for immunoprecipitation; and the third one was immunized with antibody against nucleolin. An aliquot (500 µL) of cell lysate was pre‐cleared by incubation with 200 μL of protein A/G beads on ice for 60 minutes, followed by centrifugation. Subsequently, 10 μg of anti‐nucleolin antibody was added to the pre‐cleared cell lysate, and the mixture was incubated at 4°C for 1 hours, followed by the addition of 200 μL protein A/G beads. The lysate was incubated at 4°C with agitation, and the immune complexes were separated by centrifugation at 10 000 *g* for 30 seconds at 4°C. RNA was extracted from the immunoprecipitate, and cDNA was prepared by reverse transcription with random primers and subjected to PCR.

### 2.10 Measurement of mRNA stability

Vascular smooth muscle cells were treated with either 10^−5^ mmol/L Ang II for 48 hours or nucleolin siRNA plasmid, pcDNA3.1‐Nuc plasmid or Nuc1‐309 plasmid for the indicated periods, and the cells were then incubated with either 0.5% ethanol or 5 μg/mL actinomycin D in 0.5% ethanol. Aliquots were removed from the cultures at 30‐minutes intervals over a 3‐hours time course. Actinomycin D (5 μg/mL) induced no RNA fragmentation during this period. At the indicated time‐points (0, 0.5, 1, 2 and 3 hours), 2 × 10^5^ cells were harvested, and total RNA was isolated using the Rneasy kit. PCR amplification of the pooled cDNA was carried out.

### 2.11 Synthesis of biotinylated RNA probes

The biotinylated EGF and PDGFB RNA probes were synthesized by Beijing Dingguo Changsheng Biotechnology Co., Ltd. (Table 1). The biotin‐labelled RNA probe was denatured and annealed into a double strand (program: 94°C for 5 minutes, gradually returned to room temperature), and the annealing effect was examined by 12% PAGE gel, after which biotin‐labelled RNA was diluted, packed and stored at −20°C prior to further analysis.

### 2.12 RNA‐electrophoretic mobility shift assay

For in vitro binding of RNA to proteins, 10× binding buffer [500 mmol/L KCl, 50% glycerol, 1% Non‐idet P‐40, 10 mmol/L MgCl2, 10 mmol/L dithiothreitol, and 100 mmol/L Tris–HCl (pH 8.0), 5 mg/mL heparin, and 5 mg/mL yeast tRNA] was used. The 20‐μL binding reaction system consisted of 1× binding buffer, 35 μg S100 extracts, 2 μL RNase OUT (2 U/μL) and 50 pM biotin‐labelled RNA probe. For the super‐shift assay, anti‐nucleolin (0.5 per 30 µg total protein) was added after the formation of RNA‐protein complexes. All the binding reactions were incubated at room temperature for 30 minutes, followed by addition of 2 μL protein‐loading buffer (50% glycerol, 0.02% bromophenol blue and 0.02% dimethylaniline). Samples were then separated by gel electrophoresis, transferred onto membranes and cross‐linked, and biotin‐labelled RNA was detected with the LightShift chemiluminescent EMSA kit (PIERCE).

### 2.13 Construction of recombinant plasmids pGL3‐luc‐5′UTR (EGF) and pGL3‐luc‐5′UTR (PDGF‐BB)

pGL3‐luc‐5UTR (EGF) and pGL3‐luc‐5UTR (PDGF‐BB) were constructed by Beijing Dingguo Changsheng Biotechnology Co., Ltd. as follows. Briefly, primers were designed and synthesized according to the gene sequence of interest and the vector MCS site. The EGF gene was cloned from the cDNA of rat, and the PDGFB was synthesized by whole gene. PCR was carried out using primers harbouring XhoI + HindIII and KpnI + HindIII restriction sites, fragments of about 395 and 388 bp in length were amplified, and the recovered PCR products and pGL3‐basic vector were simultaneously subjected to a double enzyme (XhoI/HindIII) digestion. In addition, the enzyme‐digested product gel was recovered and then subjected to a ligation reaction (attached with T4 ligase overnight), followed by PCR amplification. The fragment was cloned into the pGL3‐luc vector. The bacterial transformation was carried out, and the monocolonies were subjected to enzyme digestion and sequencing to confirm the successful construction of the recombinant plasmids.

### 2.14 Luciferase reporter gene assay

Cells cultured in 96‐well plates were cotransfected with the firefly‐Luciferase pGL3‐promotor vector (pGL3p; Promega) or pGL3p harbouring the 5′ UTR of EGF and PDGF‐BB, as well as the Renilla‐Luciferase phRL‐TK vector (ratio 1:5) using the Lipofectamine LTX and Plus Reagent (Invitrogen Corporation) according to the manufacturer's protocol. After 6 hours, the transfection medium was removed, and time for measurements started with the addition of fresh medium. The luciferase activity was detected using the Dual‐Glo Luciferase Assay System (Promega) and a luminometer (Labsystems Luminoscan RS) programmed with individual software (Luminoscan RII, Ralf Mrowka). The cotransfection with the Renilla‐Luciferase expression vector served as a control.

### 2.15 Statistical analysis

Data were expressed as mean ± SEM based on at least three independent experiments. Statistical analysis was performed by one‐way ANOVA (LSD test) for multiple testing. *P* < .05 was considered as statistically significant.

## 3 RESULTS

### 3.1 Bioinformatics analysis

The mRNA sequences of phenotypic transformation‐associated genes in VSMCs were screened by bioinformatics analysis, and multiple phenotypic transformation‐associated genes containing nucleolin‐binding elements ‘(T/G) CCCG (A/G)’ were identified. Bioinformatics analysis found that the mRNA sequences of 12 genes contained nucleolin‐binding elements, including EGF and PDGF‐BB.^10^ The analysis results suggested that nucleolin bound to the mRNA of these genes, regulated the expressions of these phenotype‐related genes and then modulated the phenotypic transformation of VSMCs. Recent studies have shown that EGF and PDGF‐BB play a key regulatory role in phenotype transformation of VSMCs.^16^, ^17^ Therefore, we speculated that nucleolin might bind to the mRNA of EGF and PDGF‐BB, regulate the expressions and the stability of EGF and PDGF mRNA and then positively modulate the phenotypic transformation of VSMCs.

### 3.2 Effect of Ang II on EGF and PDGF‐BB in phenotypic transformation of VSMCs

**Figure 1:**
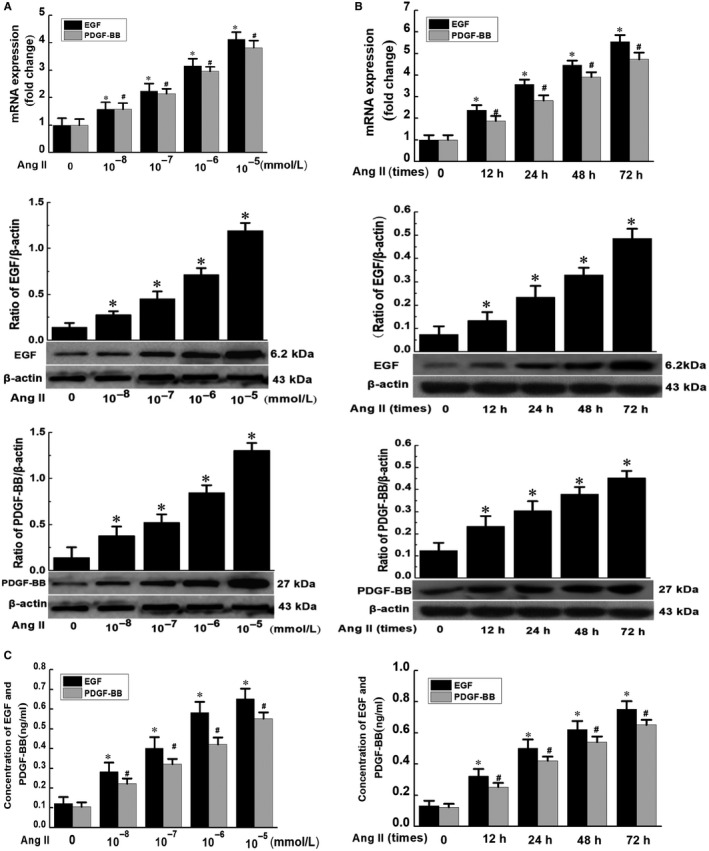
Effect of Ang II on EGF and PDGF‐BB in phenotypic transformation of VSMCs. A, VSMCs were stimulated with different concentrations of Ang II for 48 h; B, VSMCs were stimulated with Ang II (10^−5^ mmol/L) for different durations. The total RNA was extracted, and the cDNA was obtained after reverse transcription. The expressions of EGF and PDGF‐BB were detected by RT‐qPCR. The total protein was extracted, and the expressions of EGF and PDGF‐BB at the protein level were analysed by Western blotting analysis. (Data were expressed as $\bar{X}$ ± S, n = 3; * and # *P* < .05 vs the control group.) (C) After treatment of VSMCs with Ang II at different concentrations and durations, the cell culture supernatant was collected, and the secretion of EGF and PDGF‐BB was detected by ELISA. (Data were expressed as $\bar{X}$±S, n = 3; * and # *P* < .05 vs the control group)

RT‐qPCR and Western blotting analyses were used to detect the expressions of EGF and PDGF‐BB at the mRNA and protein levels in Ang II‐induced phenotype transformation of VSMCs. The results showed that the expressions of EGF and PDGF‐BB at the mRNA and protein levels in VSMCs were gradually increased after 48 hours of stimulation with different concentrations of Ang II, and the most evident effect was observed when VSMCs were treated with 10^−5^ mmol/L Ang II for 48 hours (Figure 1A). Moreover, the expressions of EGF and PDGF‐BB at the mRNA and protein levels were positively associated with the duration of Ang II exposure, and the most evident effect was observed when VSMCs were treated with 10^−5^ mmol/L Ang II for 72 hours (Figure 1B). Furthermore, ELISA results showed that the secretion of EGF and PDGF‐BB was also increased with the increase of AngII concentration and exposure duration (Figure 1C).

### 3.3 The effect of EGF and PDGF‐BB inhibition on Ang II‐induced phenotypic transformation of VSMCs

**Figure 2:**
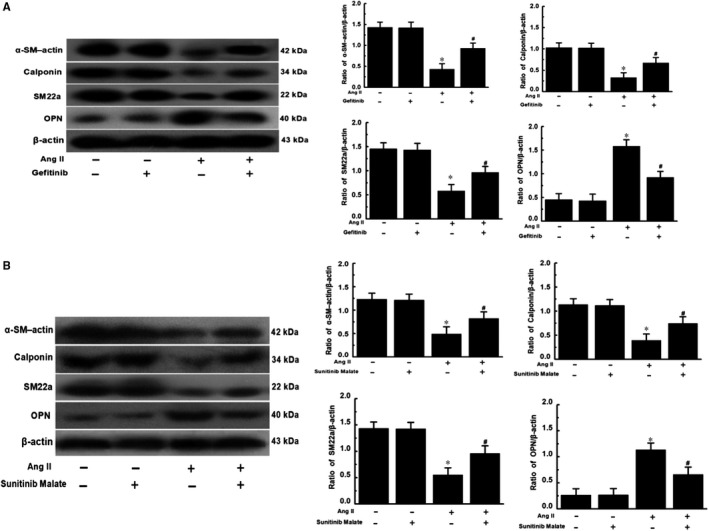
The effect of EGF and PDGF‐BB inhibition on Ang II‐induced phenotypic transformation of VSMCs. A, Effect of EGF inhibitor Gefitinib (ZD1839) on phenotypic transformation markers for phenotypic transformation of VSMCs. B, Effect of PDGF‐BB inhibitor Sunitinib Malate on phenotypic transformation markers in phenotypic transformation of VSMCs. Gefitinib (1 µmol/L) and Sunitinib Malate (2 µmol/L) were added 24 h before Ang II (10^−5^ mmol/L) treatment of VSMCs. Total protein was extracted 48 h later. The expressions of α‐SM‐actin, Calponin, SM22a and OPN at the protein level were analysed by Western blotting analysis. (Data were expressed as X ± S, n = 3; ^*^ *P* < .05 vs control group, Suntinib group and Gefitinib group; ^#^ *P* < .05 vs the Ang II group)

The above‐mentioned experiments confirmed that the expressions of EGF and PDGF‐BB at the mRNA and protein levels as well as their secretion in VSMCs were increased after Ang II treatment, confirming that EGF and PDGF‐BB played an important role in phenotypic transformation of VSMCs. To further explore the role of EGF and PDGF‐BB in the phenotypic transformation of VSMCs, we used the EGFR inhibitor Gefitinib (ZD1839) and the PDGFR inhibitor Suntinib Malate to assess the effect of Ang II on phenotypic transformation of VSMCs. The results showed that Ang II could reduce the expressions of contractile phenotype markers α‐SM‐actin, calponin and SM22a of VSMCs, and increase the expression of the synthetic phenotypic marker OPN. The EGF inhibitor Gefitinib and PDGF‐BB inhibitor Sunitinib Malate had no significant effect on the expressions of these phenotypic marker genes. However, they could significantly abolish the inhibitory effect of AngII on α‐SM‐actin, calponin and SM22a and the up‐regulation of OPN, further confirming the role of EGF and PDGF‐BB in phenotypic transformation of VSMCs (Figure 2A,B).

### 3.4 Regulatory effect of nucleolin on the expression and secretion of EGF and PDGF‐BB

**Figure 3:**
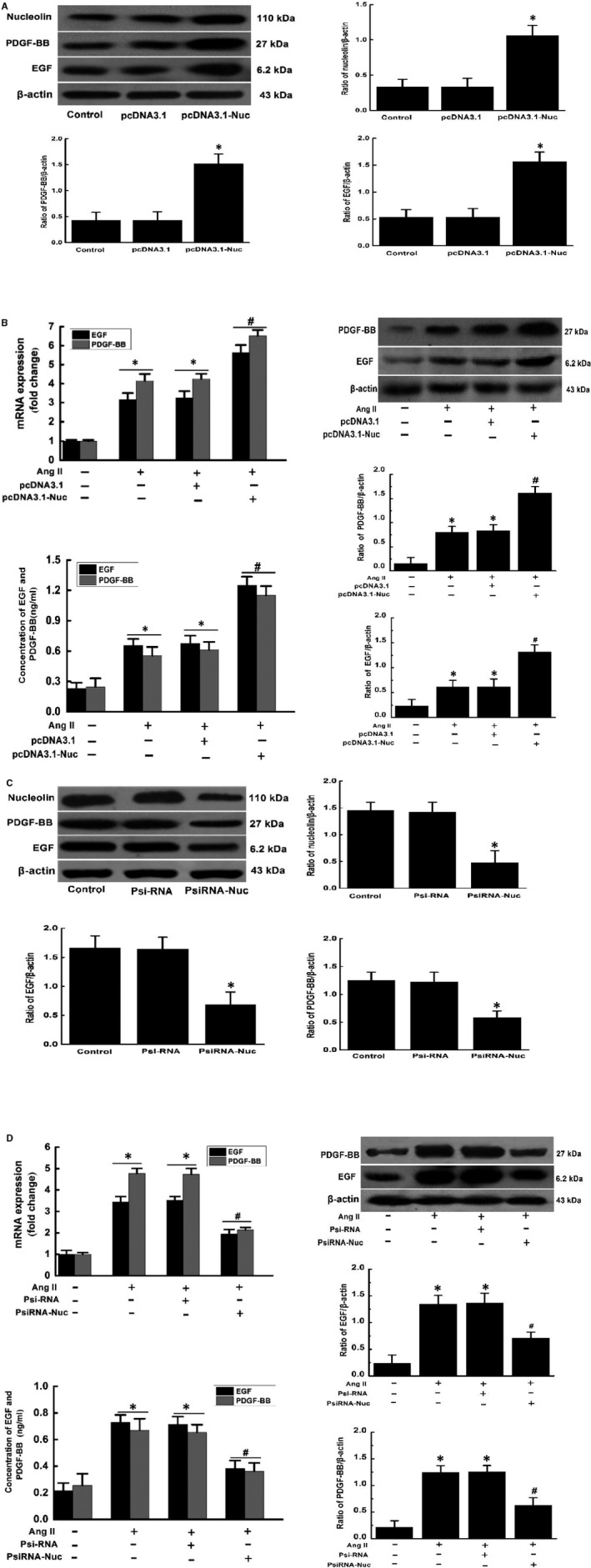
Regulatory effect of nucleolin on the expression and secretion of EGF and PDGF‐BB (A) The effect of nucleolin overexpression on the expressions of nucleolin, EGF and PDGF‐BB; (B) the effect of nucleolin overexpression on Ang II‐induced expression and secretion of EGF and PDGF‐BB. C, The effect of nucleolin low expression on expressions of nucleolin, EGF and PDGF‐BB. D, The effect of nucleolin low expression on Ang II‐induced expression and secretion of EGF and PDGF‐BB. VSMCs were transfected with the control plasmid (pcDNA3.1 or psiRNA) and the recombinant plasmid (pcDNA3.1‐Nuc or psiRNA‐Nuc), cells were treated with 10^−5^ mmol/L Ang Ⅱ for 48 h, the total protein was extracted from the transfected cells, and then RT‐qPCR, Western blotting and ELISA were performed. Ang II: AngII treatment; pcDNA3.1 and psiRNA: control plasmid; pcDNA3.1‐Nuc and psiRNA‐Nuc: nucleolin overexpression plasmid and nucleolin RNA interference plasmid, β‐actin was used as an internal control. (Data were expressed as $\bar{X}$ ± S, n = 3; ^*^ *P* < .05 vs the control group; ^#^ *P* < .05 vs the untransfected group and control plasmid group)

Previous studies have confirmed that nucleolin overexpression can promote the up‐regulation of nucleolin. After nucleolin RNA interference, the up‐regulation of nucleolin induced by AngII is significantly inhibited in VSMCs.^10^ Results demonstrated that nucleolin was overexpressed in pcDNA3.1‐Nuc‐transfected cells compared with the control plasmid pcDNA3.1 and untransfected cells. Furthermore, the expressions of EGF and PDGF‐BB at the protein level were significantly increased in pcDNA3.1‐Nuc‐transfected cells compared with the control plasmid pcDNA3.1 and untransfected cells. The expression and secretion of EGF and PDGF‐BB were increased in the Ang II‐treated group and the control plasmid pcDNA3.1 group compared with the control group following 10^−5^ mmol/L Ang II stimulation for 48 hours, while the nucleolin overexpression further increased the Ang II‐induced the expressions of EGF and PDGF‐BB at the mRNA and protein levels as well as their secretion (Figure 3A,B). In contrast, the expression of nucleolin was significantly inhibited in PsiRNA‐Nuc‐transfected cells compared with control plasmid PsiRNA group and control group. Moreover, the expressions of EGF and PDGF‐BB were significantly inhibited in PsiRNA‐Nuc‐transfected cells compared with the control plasmid pcDNA3.1 and untransfected group cells. However, Ang II‐induced expressions of EGF and PDGF‐BB at the mRNA and protein levels as well as their secretion were significantly inhibited by nucleolin RNA interference (Figure 3C,D), suggesting that nucleolin could positively regulate the expressions of EGF and PDGF‐BB at the mRNA and protein levels as well as their secretion.

### 3.5 The role of Nuc1‐309 (lacking the carboxy terminus of nucleolin) in the expression and secretion of EGF and PDGF‐BB

**Figure 4:**
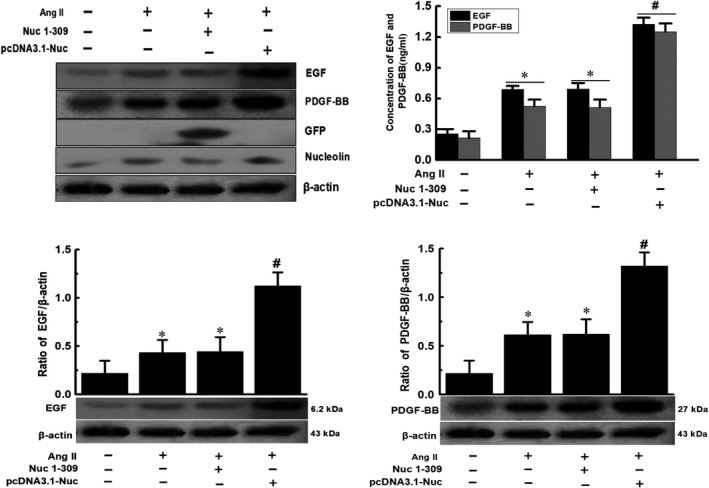
The Effect of nucleolin mutant Nuc1‐309 (lacking the carboxy terminus of nucleolin) on the expression and secretion of EGF and PDGF‐BB. VSMCs were transfected with the nucleolin mutant (Nuc1‐309) and the recombinant plasmid (pcDNA3.1‐Nuc), cells were treated with 10^−5^ mmol/L Ang II for 48 h, the total protein was extracted from the transfected cells, and then the RT‐qPCR, Western blotting and ELISA were performed. Ang II: Ang II treatment; pcDNA3.1‐Nuc: nucleolin overexpression plasmid, GFP: used to observe the nucleolin mutant (Nuc1‐309); Nucleolin: expression of nucleolin. β‐actin was used as an internal control. (Data were expressed as X ± S, n = 3; ^*^ *P* < .05 vs the control group; ^#^ *P* < .05 vs the untransfected group and Nuc1‐309 group)

The above‐mentioned results indicated that nucleolin had a regulatory effect on the expressions and secretion of EGF and PDGF‐BB, while such regulatory mechanism remained unknown. Since nucleolin is an RNA‐binding protein, we speculated that it might bind to EGF and PDGF‐BB mRNA molecules through its RBD to regulate their mRNA stability and expression. Therefore, we further used a nucleolin mutant lacking a carboxy terminus (Nuc1‐309) (ie the amino acid containing the RBD was deleted) to observe whether the nucleolin RBD still regulated the expression and secretion of EGF and PDGF‐BB. The nucleolin full‐length overexpression vector pcDNA3.1‐Nuc or the nucleolin mutant pEGFP‐N1‐Nuc1‐309 (Nuc1‐309) was transfected into VSMCs, and the results showed that the expression and secretion of EGF and PDGF‐BB were increased after Ang II treatment. Overexpression of nucleolin could significantly promote the expression and secretion of EGF and PDGF‐BB, while the overexpression of nucleolin mutant (Nucl‐309) did not promote the expression and secretion of EGF and PDGF‐BB. At the same time, the expression of nucleolin was increased after AngII treatment, and overexpression of nucleolin could significantly increase the expression of nucleolin. However, the overexpression of nucleolin mutant (Nucl‐309) could not significantly increase the expression of nucleolin (Figure 4).

### 3.6 The effect of nucleolin overexpression and low expression on the stability of EGF and PDGF‐BB mRNA

**Figure 5:**
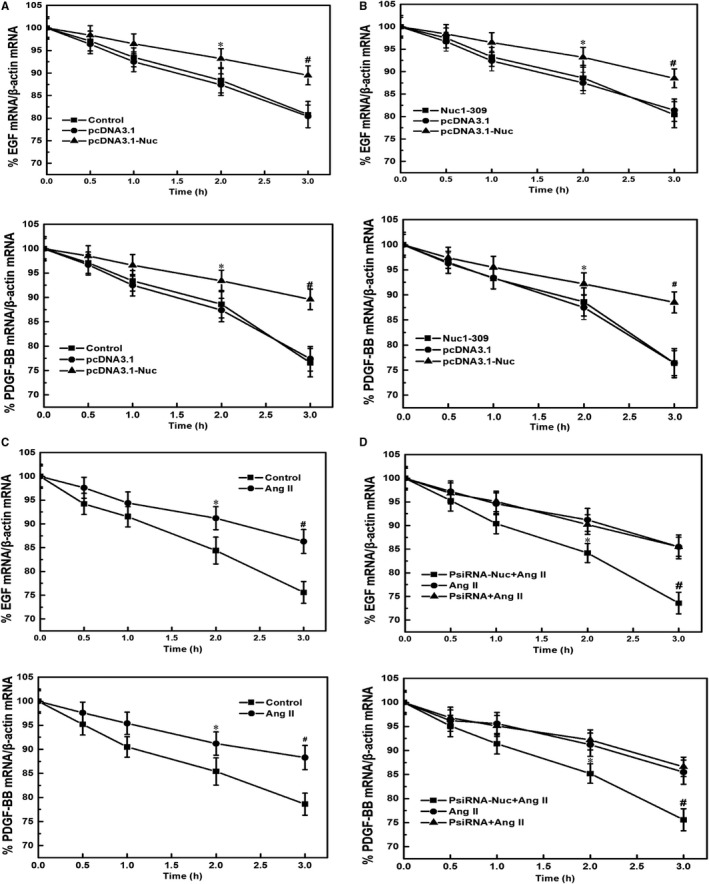
The effect of nucleolin overexpression and low expression on the stability of EGF and PDGF‐BB mRNA. A, Effect of nucleolin overexpression on the stability of EGF and PDGF. B, Effect of nucleolin mutant (Nuc1‐309) on the stability of EGF and PDGF. VSMCs were transiently transfected with nucleolin overexpression plasmid and its mutant expression plasmid for 48 h. After adding the transcription inhibitor actinomycin D (actinomycin D, 5 μg/mL), the cells were collected at different time‐points (0, 0.5, 1, 2 and 3 h), and the mRNA levels of EGF and PDGF were detected by RT‐qPCR. Control: normal VSMCs; pcDNA3.1: control plasmid group; pcDNA3.1‐Nuc: nucleolin overexpression plasmid group; Nuc1‐309: nucleolin mutant plasmid lacking the carboxy terminus of nucleolin (ie deleting the amino acid containing the RNA‐binding domain); β‐actin was used an internal control (Data were expressed as X ± S, n = 5; ^*^ *P* < .05, ^#^ *P* < .01 vs the pcDNA3.1 and Nuc1‐309 group.) (C) Effect of Ang II on the stability of EGF and PDGF. After treatment of VSMCs with 10^−5^ mmol/L Ang II for 48 h, cells were harvested at different time‐points (0, 0.5, 1, 2 and 3 h) after adding the transcription inhibitor actinomycin D (5 μg/mL), and the mRNA levels of EGF and PDGF were detected by RT‐qPCR. Control: normal VSMC group; Ang II: Ang II treatment group; β‐actin was internal control (Data were expressed as X ± S, n = 5; ^*^ *P* < .05, ^#^ *P* < .01 vs the control group). D, Effect of low expression of nucleolin on the stability of EGF and PDGF induced by Ang II. After VSMCs were transfected with nucleolin interference plasmid for 24 h, VSMCs were treated with 10^−5^ mmol/L Ang II for 48 h, cells were harvested at different time‐points (0, 0.5, 1, 2 and 3 h) after adding the transcription inhibitor actinomycin D (5 μg/mL), and the mRNA levels of EGF and PDGF were detected by RT‐qPCR. Ang II: Ang II treatment group; PsiRNA: control plasmid; PsiRNA‐Nuc: nucleolin RNA interference plasmid. β‐actin was used as an internal control (Data were expressed as X ± S, n = 5; ^*^ *P* < .05, ^#^ *P* < .01 vs the PsiRNA and Ang II‐treated group)

The results showed that the expressions of EGF and PDGF‐BB at the mRNA level were gradually decreased after the treatment of transcription inhibitor actinomycin D (5 μg/mL) in normal cells. Compared with the control group, the overexpression of nucleolin slowed down the degradation of EGF and PDGF‐BB mRNA, indicating increased stability of EGF and PDGF‐BB mRNA. However, the control plasmid pcDNA3.1 had no significant effect on the stability of EGF and PDGF‐BB mRNA. Moreover, transfection of the nucleolin mutant (Nucl‐309) did not increase the stability of EGF and PDGF‐BB mRNA (Figure 5A,B). The effect of Ang II on the stability of EGF and PDGF‐BB mRNA was also further analysed. Compared with the control group, Ang II slowed down the degradation of EGF and PDGF‐BB mRNA, indicating increased stability of EGF and PDGF‐BB mRNA. VSMCs were transfected with nucleolin siRNA for 24 hours before Ang II treatment, and the effect of Ang II on the stability of EGF and PDGF‐BB mRNA was relieved. However, the control plasmid PsiRNA had no significant effect on the stability of EGF and PDGF‐BB mRNA (Figure 5C,D).

### 3.7 Binding of nucleolin protein to EGF and PDGF‐BB mRNA

**Figure 6:**
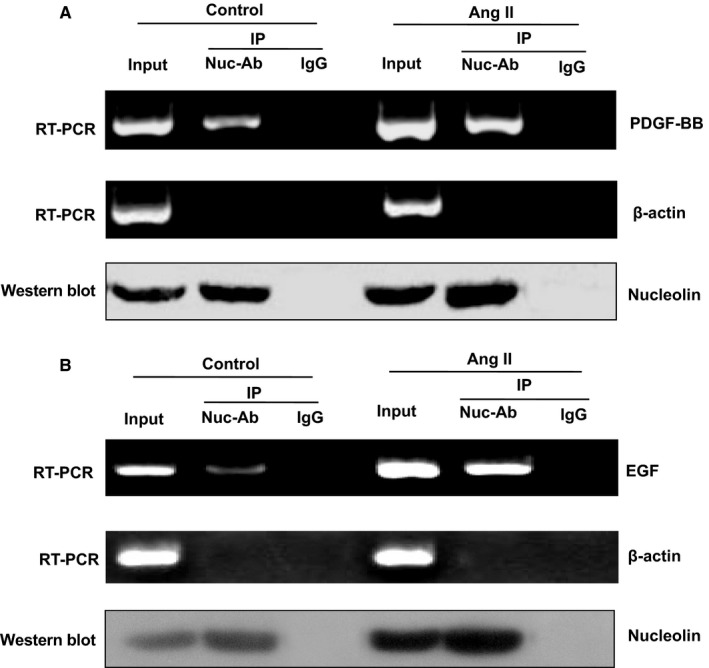
The binding of nucleolin protein with EGF and PDGF‐BB mRNA in VSMCs. A, Binding of nucleolin protein to PDGF‐BB mRNA; B, binding of nucleolin protein to EGF mRNA Immunoprecipitation and RT‐PCR were used to analyse the combination of nucleolin and EGF, PDGF‐BB mRNA. Normal VSMCs and Ang II‐treated VSMCs were collected to prepare the cell extracts, and cell extracts were divided into three equal groups as follows: Input group, negative control IgG group and nucleolin antibody group. The nucleolin antibody was used for co‐immunoprecipitation, and the total RNA in half of the precipitate was extracted. The EGF, PDGF‐BB and β‐actin specific primers were used for RT‐qPCR detection, and the other half of the precipitate was detected by Western blotting analysis for the amount of nucleolin. Representatives of three separate experiments. RT‐qPCR, reverse transcription‐quantitative polymerase chain reaction; Ang II, angiotensin II treatment (10^−5^ mmol/L Ang II for 48 h); Input, positive control; IgG, immunoglobulin G negative control; Nuc‐Ab, nucleolin antibody; Ctrl, control cells; IP, immunoprecipitation

To confirm the presence of interaction between nucleolin and EGF or PDGF‐BB mRNA, we used protein immunoprecipitation and RT‐qPCR to verify their interaction. Results showed that only a small amount of EGF and PDGF‐BB mRNA was precipitated in normal cell lysate. However, the amount of EGF and PDGF‐BB mRNA precipitated by nucleolin antibody was increased compared with the control IgG group, suggesting that nucleolin could bind to EGF and PDGF‐BB mRNA under the normal condition. The amount of EGF and PDGF‐BB mRNA in cell lysate was significantly increased after Ang II stimulation. After co‐immunoprecipitation with nucleolin antibody, the EGF and PDGF‐BB mRNA precipitated by nucleolin antibody were significantly increased compared with the control IgG group, suggesting that the binding ability of nucleolin to EGF and PDGF‐BB mRNA molecules was increased after VSMCs were treated with Ang II. We found that the binding of nucleolin to EGF and PDGF‐BB mRNA molecules was specific using β‐actin as a control. Western blotting analysis was used to detect the content of nucleolin in the cell extracts and sediments, which confirmed the effectiveness of the nucleolin antibody (Figure 6A,B).

### 3.8 Binding of nucleolin to the 5' UTR of EGF and PDGF‐BB mRNA

**Figure 7:**
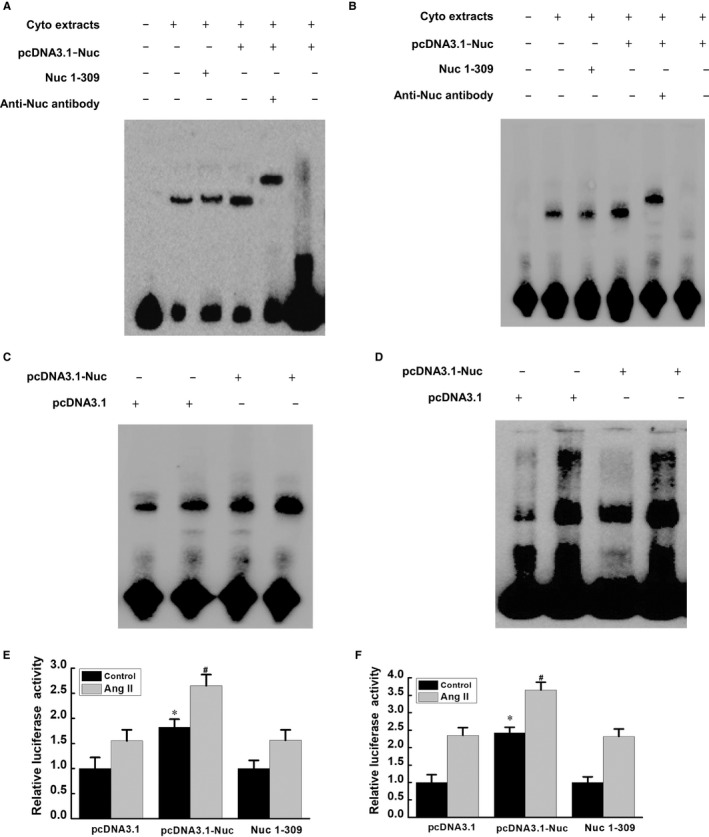
RNA‐EMSA and Luciferase reporter gene assay of the binding of nucleolin to the 5′ UTR of EGF and PDGF‐BB mRNA. A, The binding of nucleolin to the 5′ UTR of EGF mRNA. B, The binding of nucleolin to the 5′ UTR of PDGF‐BB mRNA. Cyto extracts: cytoplasmic protein; pcDNA3.1: control plasmid; pcDNA3.1‐Nuc: overexpression plasmid; Nuc1‐309: nucleolin mutant with lacking the carboxy terminus of nucleolin (ie deleting the amino acid containing the RNA‐binding domain); anti‐nuc antibody: nucleolin antibody; biotin‐labelled probe: biotin‐labelled EGF 5′ UTR RNA probe or biotin‐labelled PDGF 5′ UTR RNA probe; 100‐fold competition probe: 100‐fold unlabelled probe; C, effect of Ang II on the binding activity of nucleolin and EGF mRNA; D, effect of Ang II on the binding activity of nucleolin and PDGF‐BB mRNA. Representatives of three separate experiments. Ang II: AngII treatment (10^−5^ mmol/L Ang II for 48 h); E, Luciferase reporter gene assay for the regulation of EGF mRNA 5′ UTR by nucleolin; F, Luciferase reporter gene assay for the regulation of nucleolin on PDGF‐BB mRNA 5′ UTR. Nuc1‐309: Nuc1‐309: nucleolin mutant with lacking the carboxy terminus of nucleolin (ie deleting the amino acid containing the RNA‐binding domain); Control: normal cell control group (ie no Ang II treatment group); Ang II: Ang II treatment (10^−5^ mmol/L Ang II for 48 h); pcDNA3.1‐Nuc: overexpression plasmid; pcDNA3.1: control plasmid; ^*^ *P* < .05 vs pcDNA3.1 and Nuc1‐309 group, ^#^ *P* < .05 vs pcDNA3.1 and Nuc1‐309 group

Preliminary results confirmed that nucleolin could bind to the mRNA of EGF and PDGF‐BB. However, it remained unclear how nucleolin bound to EGF and PDGF‐BB mRNA and then regulated their stability. Based on bioinformatics analysis, we speculated that nucleolin bound to the 5′ UTR of EGF and PDGF‐BB mRNA to increase their stability. We extracted the cytoplasmic proteins of normal VSMCs, nucleolin over‐expressing and mutant (Nuc 1‐309) over‐expressing VSMCs and used RNA‐EMSA to detect the binding of nucleolin to 5′ UTR of EGF and PDGF‐BB mRNA. The results showed that the probe retention signal (probe binding band) was observed in normal VSMCs, and the probe binding band was significantly enhanced after overexpression of nucleolin. The binding band of nucleolin mutant Nuc1‐309 was not significantly enhanced. When the non‐biotinylated EGF and the PDGF‐BB 5′ UTR RNA probe (100‐fold concentration) competed, the binding band disappeared. If the nucleolin antibody was added to the reaction system, the signal of the original binding band was significantly weakened, and a supermigration band appeared above the binding band (Figure 7A,B). This result suggested that nucleolin could bind to the 5′ UTR of EGF and PDGF‐BB mRNA. In order to observe the effect of Ang II treatment on the binding activity of nucleolin to EGF and PDGF‐BB mRNA, the nuclear proteins of nucleolin overexpression and control plasmids were extracted, respectively. The binding of nucleolin to EGF and PDGF‐BB mRNA was detected by EMSA. The results showed that after Ang II treatment, the binding activity of nucleolin to EGF and PDGF‐BB mRNA in control plasmid and nucleolin over‐expressing groups was significantly higher compared with that without Ang II treatment, and the binding ability of nucleolin over‐expressing group was stronger compared with the control plasmid (Figure 7C,D).

The above‐mentioned results confirmed that nucleolin could enhance the stability of EGF and PDGF‐BB mRNA and could bind to the 5′ UTR of EGF and PDGF‐BB mRNA. However, it still remained unknown whether such binding enhanced the stability of EGF and PDGF‐BB mRNA and then regulated the expressions of EGF and PDGF‐BB. Therefore, we constructed a pGL3‐promoter recombinant plasmid containing EGF and PDGF‐BB mRNA 5' UTR and used luciferase reporter gene system to observe the regulatory mechanism of nucleolin on EGF and PDGF‐BB mRNA 5′ UTR. The results showed that the activity of luciferase was increased in the Ang II‐treated group compared with the normal control group. Meanwhile, the activity of the luciferase in nucleolin over‐expressing group was increased compared with the control plasmid pcDNA3.1 group. However, there was no significant change in the activity of luciferase in the nucleolin mutant Nuc1‐309 plasmid group (Figure 7E,F). This result suggested that nucleolin had a positive regulatory effect on the 5′ UTR of EGF and PDGF‐BB mRNA.

## 4 DISCUSSION

Our previous studies have shown that nucleolin expression is up‐regulated and nucleolin is shifted from the nucleus to the cytoplasm after VSMCs are stimulated with Ang II at different concentrations and durations. This finding suggests that nucleolin also plays a role in Ang II‐induced phenotype transformation of VSMCs, moreover, it may function depending on its cytoplasmic localization. Furthermore, nucleolin overexpression significantly promoted Ang II‐induced phenotypic transformation of VSMCs. After down‐regulation of nucleolin expression, its promotive effect on phenotypic transformation was abolished, confirming that nucleolin played a positive role in Ang II‐mediated phenotype transformation of VSMCs.^10^ These results confirmed that up‐regulation of nucleolin played an important regulatory role in the phenotypic transformation of VSMCs induced by Ang II. However, what is the mechanism of action of nucleolin remains unclear. As an RNA‐binding protein, whether nucleolin binds to the mRNA of VSMCs phenotype‐transformed genes, regulates their stability and expression, and then plays a role in phenotype transformation of VSMCs? In other words, whether nucleolin can regulate the expressions of phenotypic transformation‐related factors of VSMCs at the post‐transcriptional level, thereby regulating VSMCs phenotypic transformation? The phenotypic transformation of VSMCs is currently known to be affected and regulated by a variety of growth factors and cytokines, such as PDGF, EGF, fibroblast growth factor (FGF) and insulin‐like growth factors (IGF‐1). In addition, phenotypic transformation of VSMCs is also affected and regulated by vasoactive substances (Ang II), tumour necrosis factor‐alpha (TNF‐α), extracellular matrix (ECM) and matrix metalloproteinase (MMP).^3^, ^4^, ^17^, ^18^, ^19^, ^20^, ^21^, ^22^, ^23^ The effects of these extracellular signalling molecules on the corresponding receptors of VSMCs can be transferred into the cells by multiple intracellular signalling pathways, and the phenotypic transformation process of the cells is initiated by inducing the expression of cyclin gene. Firstly, according to bioinformatics analysis, nucleolin might bind to the mRNA of EGF and PDGF‐BB, regulate the expressions of EGF and PDGF‐BB, and then modulate the phenotypic transformation of VSMCs. Recent studies have shown that EGF and PDGF‐BB play a key regulatory role in phenotype transformation of VSMCs, Therefore, we speculated that nucleolin might bind to the mRNA of EGF and PDGF‐BB, regulate the expressions of these genes, and play an important role in the phenotypic transformation of VSMCs. PDGF‐BB is one of the earliest factors that can promote the phenotypic transformation of VSMCs. It is a strong mitogenic factor with biological activity that promotes proliferation and migration, and enhances the synthesis and secretion of VSMCs. PDGF‐BB can induce expression or silencing of specific DNA, thereby stimulating phenotypic transformation and proliferation of VSMCs. PDGF‐BB can bind to all PDGF receptors, phosphorylate receptors, and activate important downstream signalling molecules. It plays a role in promoting phenotypic transformation of VSMCs mainly through MEK1/ERK and MKK6/p38MAPK pathways. Inhibition of PDGF‐BB can significantly inhibit phenotypic transformation, proliferation and migration of VSMCs, thereby reducing the formation of angiogenic intima.^24^, ^25^ Similarly, EGF is also one of the most important cytokines known to promote proliferation and migration of VSMCs. As an important cell growth factor, EGF binds to its specific receptor (EGFR) and activates a series of cell signalling pathways, playing an important role in phenotypic transformation, proliferation and differentiation of VSMCs.^26^ Studies have shown that EGF and EGFR‐mediated signalling pathways play a very important role in remodelling of blood vessel walls and neointimal formation after vascular injury. Moreover, the inhibition of atherosclerosis by suppressing EGF signalling has been validated in different animal models.^27^, ^28^ In order to investigate whether nucleolin played a role in the phenotypic transformation of VSMCs by binding to the mRNA molecules of EGF and PDGF‐BB, regulated their stability and up‐regulated their expressions, we first observed the expressions of EGF and PDGF‐BB at the mRNA and protein levels in the phenotype transformation of VSMCs induced by Ang II. The results showed that the expression and secretion of EGF and PDGF‐BB were gradually increased in VSMCs after 48 hours of Ang II treatment at different concentrations and durations, suggesting that EGF and PDGF‐BB played an important role in phenotype transformation of VSMCs. To further confirm the role of EGF and PDGF‐BB in the phenotypic transformation of VSMCs, we used the EGFR inhibitor Gefitinib and the PDGFR inhibitor Sunitinib to assess the effect of Ang II on phenotypic transformation of VSMCs. The results showed that the above‐mentioned inhibitors significantly abolished the promotive effect of Ang II on phenotypic transformation of VSMCs. This result confirmed that EGF and PDGF‐BB played an important role in the phenotypic transformation of VSMCs induced by Ang II, which was consistent with previous literature reports.^29^ Furthermore, we explored the regulatory effects of nucleolin on EGF and PDGF‐BB. The results showed that overexpression of nucleolin could up‐regulate the expression and secretion of EGF and PDGF‐BB, while the expression and secretion of EGF and PDGF‐BB were significantly inhibited after nucleolin RNA interference, suggesting that nucleolin had a positive regulatory effect on the expression and secretion of EGF and PDGF‐BB. Studies have shown that the RNA‐binding properties of nucleolin play a fundamental role in regulating various biological functions, such as cell growth and proliferation. As an RNA‐binding protein, it remains unclear whether nucleolin regulates the expression and secretion of EGF and PDGF‐BB through its RBD. Further studies using nucleolin mutants revealed that nucleolin mutant lacking a carboxy terminus (Nuc1‐309) lost its regulatory effect on the expressions of EGF and PDGF‐BB. This finding suggested that the regulatory effect of nucleolin on EGF and PDGF‐BB expression was likely dependent on the presence of its RBD. The regulation of gene expression mainly includes the regulation of transcriptional level, post‐transcriptional level, translational level and post‐translational level. The regulation of translational level mainly includes regulation of translation initiation and regulation of mRNA stability. Moreover, mRNA is a template for translation of proteins, the amount of which directly affects the amount of protein synthesis. Therefore, the stability of mRNA plays an important role in the regulation of translation. Post‐transcriptional regulation usually involves the interaction of a specific sequence of the 5′ or 3′ UTR of a mature mRNA with a corresponding protein, which mediates nuclear export of mRNA, localization in the cytoplasm, initiation of translation and degradation of mRNA, thereby regulating the expression level of the gene. For example, nucleolin binds to the 5′ UTR of interleukin (IL) 2 mRNA and growth arrest and DNA damage inducible α (GADD45α) mRNA coding region and regulates the expressions of target proteins by modulating the stability of mRNA of these target genes.^30^, ^31^ In addition, the RBD of nucleolin can also bind to the 5′ or 3′ UTR of mRNA of various apoptosis‐related genes, such as Bcl‐2, AKT1 and p53, by which the corresponding mRNA is stabilized or destabilized, thereby promoting cell proliferation and anti‐apoptotic effects.^11^, ^12^, ^32^ We analysed the mRNA sequences of EGF and PDGF‐BB by bioinformatics analysis and found that the 5′ UTR in the EGF and PDGF‐BB mRNA sequences contained nucleolin‐specific binding elements. Therefore, it is necessary to investigate whether the RNA‐binding protein nucleolin could bind to the 5′ UTR of EGF and PDGF‐BB mRNA to regulate the stability of EGF and PDGF‐BB mRNA.

In this study, the transcriptional inhibitor actinomycin D was used to inhibit the transcription of genes, and the effects of Ang II, nucleolin overexpression, nucleolin low expression and Nucl‐309 on the stability of EGF and PDGF‐BB mRNA were observed. The results showed that Ang II could slow down the degradation of EGF and PDGF‐BB mRNA, and nucleolin could increase the stability of EGF and PDGF‐BB mRNA. However, transfection of the nucleolin mutant (Nucl‐309) did not increase the stability of EGF and PDGF‐BB mRNA. It remained unclear how nucleolin regulated the stability of EGF and PDGF‐BB mRNA. In this study, we first found that nucleolin could bind to EGF and PDGF‐BB mRNA in vivo by IP‐RT‐PCR. Furthermore, we found by RNA‐EMSA that nucleolin could bind to the 5′ UTR of EGF and PDGF‐BB mRNA in vitro, and the binding activity of nucleolin to EGF and PDGF‐BB mRNA was significantly increased under the stimulation of Ang II. Moreover, we also found that nucleolin could also bind to EGF and PDGF‐BB mRNA under physiological conditions. However, what was the specific effect of nucleolin on the phenotypic transformation of VSMCs under physiological conditions was not clear. Nucleolin may play different functions in different cell environments, and further research is needed. To further demonstrate whether the binding of nucleolin to the 5′ UTR of EGF and PDGF‐BB mRNA affected the stability and expression of EGF and PDGF‐BB mRNA, we constructed a luciferase reporter gene containing EGF or PDGF‐BB mRNA 5′ UTR. The reporter gene was cotransfected with nucleolin over‐expressing plasmid into VSMCs and analysed by dual fluorescent reporter gene detection system. The results showed that the binding of nucleolin to the 5′ UTR of EGF and PDGF‐BB mRNA could up‐regulate the stability and expression of EGF and PDGF‐BB mRNA, suggesting that nucleolin had a positive regulatory effect on the 5′ UTR of EGF and PDGF‐BB mRNA. Although this study clarified that nucleolin delayed the degradation of EGF and PDGF‐BB mRNA and increased the stability of EGF and PDGF‐BB mRNA, it remained unknown how nucleolin binding to EGF and PDGF‐BB mRNA 5' UTR increased the stability of EGF and PDGF‐BB.

## 5 CONCLUSIONS

In conclusion, our current study demonstrated that nucleolin increased the stability of EGF and PDGF‐BB mRNA and up‐regulated their expressions by binding to the mRNA of EGF and PDGF‐BB, thereby promoting the Ang II‐induced phenotypic transformation of VSMCs. However, the relationship between nucleolin and the mRNA of other phenotype‐related genes remains unexplored. Moreover, whether nucleolin can regulate the genes involved in the inhibition of phenotypic transformation of VSMCs is still unclear. In addition, research has shown that nucleolin has DNA‐binding properties and works with other proteins to play a role in transcriptional regulation of genes. The nucleolin and ribonucleoprotein C bind to the promoter of the pre‐amylase (APP) gene and mediate the transcription of the APP gene.^33^ Next, further study is required to explore whether nucleolin can bind to the promoter region of phenotypic transformation‐associated genes, such as EGF and PDGF‐BB, and regulate the phenotypic transformation of VSMCs at the transcriptional level. The phenotypic transformation process of VSMCs is extremely complex, and our study was likely to provide a new perspective for the regulation of phenotypic transformation of VSMCs. Nucleolin might play a role in promoting phenotypic transformation by mediating post‐transcriptional regulation of VSMC phenotypic transformation‐related genes, affecting the stability and protein expression of related genes. Therefore, nucleolin was also likely to become a new drug intervention target for cardiovascular disease treatment, such as hypertension, atherosclerosis and restenosis after angioplasty. Besides, our findings provided a theoretical basis for the development of new drugs that regulate phenotypic transformation, proliferation and differentiation of VSMCs.

## ETHICAL APPROVAL STATEMENT

The procedures in the study were scrutinized and approved by Medical Ethics Committee of Xiangya Hospital, Central South University.

## CONFLICTS OF INTEREST

The authors declare no conflict of interest.

## AUTHOR CONTRIBUTIONS

All authors contributed extensively to the work presented in this paper. LF performed the all experiments, analysed statistical data and wrote and revised the manuscript. Z.‐XY designed the current study, acquired data, analysed and interpreted the data. K.‐KW and P.‐FZ participated in the design, analysed the data and revised the manuscript. TL performed the experiments and analysed data. Z.‐LX and M.Y acquired and analysed the data. All authors read and edited the manuscript and approved the final version.

## Data Availability

The data that support the findings of this study are available from the corresponding author upon reasonable request.
